# deMix: Decoding Deuterated Distributions from Heterogeneous Protein States via HDX-MS

**DOI:** 10.1038/s41598-019-39512-8

**Published:** 2019-02-28

**Authors:** Seungjin Na, Jae-Jin Lee, Jong Wha J. Joo, Kong-Joo Lee, Eunok Paek

**Affiliations:** 1 Dept. of Computer Science, Hanyang University, Seoul, 04763 South Korea; 2 Graduate School of Pharmaceutical Sciences, College of Pharmacy, Ewha Womans University, Seoul, 03760 South Korea; 3 Dept. of Computer Science and Engineering, Dongguk University-Seoul, Seoul, 04620 South Korea

## Abstract

Characterization of protein structural changes in response to protein modifications, ligand or chemical binding, or protein-protein interactions is essential for understanding protein function and its regulation. Amide hydrogen/deuterium exchange (HDX) coupled with mass spectrometry (MS) is one of the most favorable tools for characterizing the protein dynamics and changes of protein conformation. However, currently the analysis of HDX-MS data is not up to its full power as it still requires manual validation by mass spectrometry experts. Especially, with the advent of high throughput technologies, the data size grows everyday and an automated tool is essential for the analysis. Here, we introduce a fully automated software, referred to as ‘deMix’, for the HDX-MS data analysis. deMix deals directly with the deuterated isotopic distributions, but not considering their centroid masses and is designed to be robust over random noises. In addition, unlike the existing approaches that can only determine a single state from an isotopic distribution, deMix can also detect a bimodal deuterated distribution, arising from EX1 behavior or heterogeneous peptides in conformational isomer proteins. Furthermore, deMix comes with visualization software to facilitate validation and representation of the analysis results.

## Introduction

Analysis of a protein structure and its molecular composition is essential for understanding the protein’s underlying function. While we believe that a sequence of amino acids encodes a precise three-dimensional shape of a protein, characterizing protein structures from primary sequences is still a challenge. Recently, combining amide hydrogen/deuterium exchange (HDX) with mass spectrometry (MS) is favored as a tool for characterizing dynamic structure of proteins. HDX is a chemical reaction in which a covalently bonded hydrogen atom is replaced by a deuterium atom, or vice versa^1^. The HDX provides structural information based on solvent accessibility of peptide bonds in tertiary and quaternary structure of each protein, because exchange rates between hydrogen and deuterium in proteins depend on the degree of a proton’s exposure to the protein surface and flexibility of the surrounding tertiary structure in permitting access of protons to solvent.

X-ray crystallography provides detailed structural information of a protein if appropriate crystals can be generated as a solid state. However, often crystal structures of all proteins are unavailable. Moreover, X-ray crystallography provides only the static structure of a crystal but not a dynamic view of a protein in the solution. Alternatively, high field nuclear magnetic resonance (NMR) can be used to obtain the information about the structure and dynamics of a protein. Unfortunately, it requires high concentrations of sample^2^, which possibly cause the distortion and aggregation of a protein structure. Besides, there are several other analytical tools such as circular dichroism, fluorescence, differential scanning calorimetry, analytical ultracentrifugation, side-chain reactivity, binding assays, and various chromatographic methods available and commonly used for protein biophysical characterization^3^. However, none of these methods provides an easy or straightforward manner to determine the entire structure of a protein without the help of experts. For the reasons, there have been efforts to combine multiple tools together. HDX-MS has many apparent advantages compared to NMR or X-ray crystallography: it is possible to analyze proteins in native solution condition; it requires much less material for analysis due to growing sensitivity of a mass spectrometer; moreover, protein mixtures are also compatible to analysis. Especially, HDX-MS can provide information for active dynamic structural changes of a protein under various biological conditions.

The main computational problem in HDX-MS analysis is to determine deuterium contribution to isotopic distribution of a deuterated peptide. There are several methods for calculating the number of exchanged deuteriums. One approach to obtaining an average deuteration level is to simply calculate a centroid mass of a deuterated isotopic distribution and subtract the centroid mass of the corresponding natural (non-deuterated) isotopic distribution^4–8^. A more sophisticated approach is to solve linear equations, where the deuterated isotopic distribution is defined as convolution of its natural isotopic distribution and the distribution for the deuteration level of the peptide^9–12^. The best fit to all of the linear equations is achieved using least-squares method^9^ or maximum entropy method (MEM)^10^. The least-squares method is simple but not applicable when the number of exchangeable hydrogen is big or signal-to-noise ratio is poor. MEM uses entropy to measure the amount of uncertainty in a probability distribution and finds the highest entropy solution subject to the error range. MEM requires substantial computation and can be unreliable when MEM spectral distortion occurs, but it is generally considered to be more robust to the noise than other methods. Fourier deconvolution method^11,12^ uses Fast Fourier Transform for deconvolution of natural and deuterated isotopic distributions, consequently revealing the deuteration level.

One of the difficulties in the determination of deuterium contribution is that often a deuterated isotopic distribution has a bimodal form, not a single form, which arises from EX1 behavior or heterogeneous conformational populations^13^. The kinetics for HDX has two limitations, EX2 and EX1^14,15^. In EX2 condition, a progressive mass shift in single deuterated distribution is observed with increasing D_2_O labeling time, while in EX1 condition, a progressive amount reweighting between two deuterated distributions is observed. More interestingly, the coexistence of two protein conformations may lead to a bimodal deuterated distribution, where simultaneous, progressive mass shifts in two distributions can be observed with increasing D_2_O labeling time. Nevertheless, most of the previous HDX software assume only the unimodal distribution, resulting in a single deuterium number. For example, centroid mass-based approaches ignore the shape of the observed deuterated distribution and determine the (single) center of masses of all observed peaks. Some software tools such as ExMS^16^, Hexicon 2^17^ and HX-Express v2^18^ have been proposed to identify a bimodal isotopic distribution for the deuterated distribution.

Here, we introduce a new algorithm, referred to as ‘deMix’ (decode deuterated mixture) that can analyze HDX-MS data in a fully automated fashion, which is essential for avoiding human errors, while allowing for high throughput data analysis. In particular, deMix makes it possible to interpret the bimodal deuterated distributions, reporting deuterium numbers up to two. Basically, deMix assumes a statistical distribution of deuterium contribution to a deuterated peptide as the binomial distribution, and deals directly with the shape of deuterated isotopic distribution, but not considering their centroid masses. Deuterated isotopic distribution naturally spans a wider *m/z* range than the corresponding natural isotopic distribution, due to partial deuteration. Thus, there can be more noises and frequent overlaps between isotopic distributions in HDX-MS than ordinary MS data, which complicates the recognition of a peptide’s isotope pattern and calculation of the deuteration level. To overcome such difficulties, deMix proposes a measure, referred to as *Matched Peak Count*, which is designed to be robust over random noises in comparing two distributions. In addition, the proposed measure has strength in analyzing bimodal deuterated distributions. Applied to HDX-MS experiments with native and oxidized Nm23-H1, a tumor metastasis suppressor, deMix could accurately predict the deuteration level not only when the deuterated isotopic distribution has a single deuterated form but also for when it has a bimodal form as two proteins coeluted in a MS run. Finally, deMix comes with a visualization software to facilitate validation and representation of the analysis results, offering greater practical utility.

## Results

### The deMix algorithm

**Figure 1:**
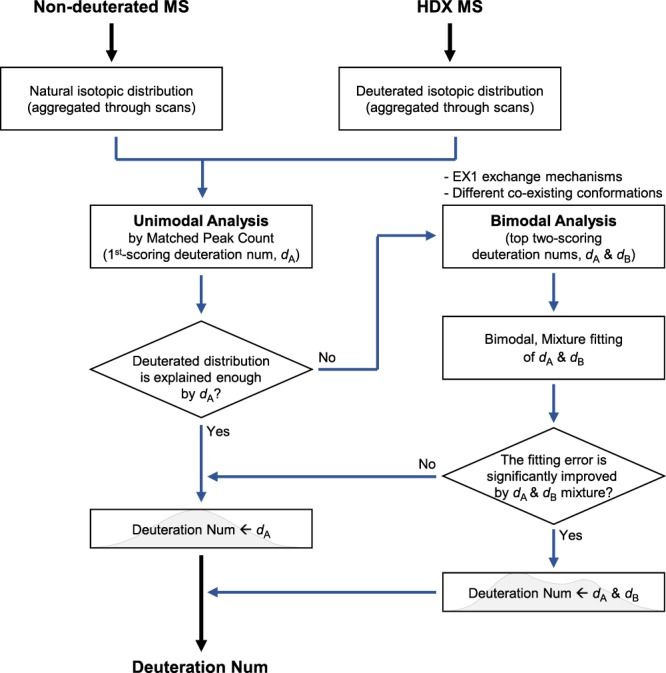
The deMix workflow.

**Figure 2:**
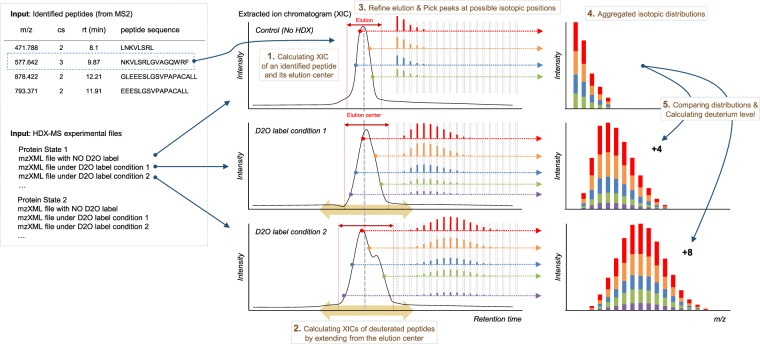
The detection of natural and deuterated isotopic distributions of peptides. Isotopic distributions on XIC of a peptide are aggregated into a representative distribution.

The deMix workflow is summarized in Fig. 1. deMix determines the H/D exchange profile via binomial fitting and decides whether to perform bimodal analysis without any human intervention. The analysis starts with detecting natural and deuterated isotopic distributions of peptides of interest and the procedure is shown in Fig. 2: (1) given peptides identified from MS2, deMix generates a theoretical isotopic distribution for each peptide and compares observed distributions in MS1 spectra (non-deuterated sample), constructing its extracted ion chromatogram (XIC). More details are provided under Methods; (2) based on the XIC in the non-deuterated sample, XICs of peptides in deuterated samples are constructed, where our assumption is that the related XICs across samples partially overlap or are shifted within a certain range (e.g., ±40 scans) although they may not totally overlap; (3) deMix refines each XIC and selects isotopic distribution peaks corresponding to presumably the same peptide ion within a determined elution time span; (4) deMix aggregates all detected isotopic peaks into a single isotopic distribution. Individual isotopic distributions typically have poor shape when the intensities of peaks are not high enough or a few isotopic distributions overlap and the similarity of the individual distributions became weak. The aggregated isotopic distributions is regarded more robust than individual distributions; and (5) based on the aggregated isotopic distributions, deuterium numbers are determined. The algorithm is described in the next section.

### Fundamental algorithm to analyze deuterated distributions

**Figure 3:**
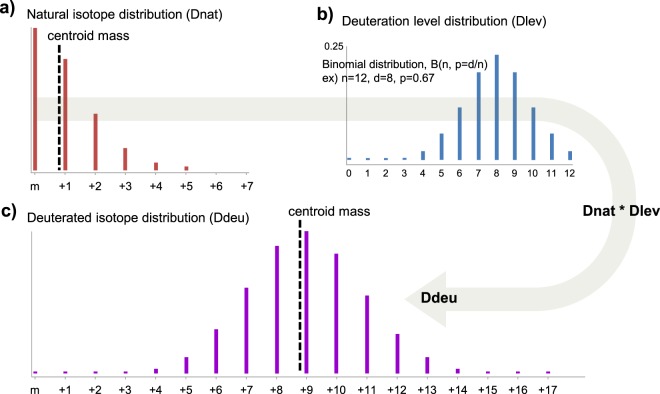
Three distributions in HDX-MS data. In this example, among 12 exchangeable hydrogens of a peptide, 8 are deuterated on average. A peptide is deuterated following binomial behavior, where each of *n* exchangeable hydrogens in a peptide is statistically deuterated with identical labeling probability *p*, which is calculated by dividing the average deuterium number *d* by *n*. As a result, observed, deuterated isotopic distribution is represented as the convolution of natural isotopic distribution and statistical deuteration distribution. The dotted line represents the centroid mass (CM) of the distribution. The centroid mass-based methods calculate the average deuterium number as CM(Ddeu) minus CM(Dnat).

HDX-MS data have to do with three relevant distributions as shown in Fig. 3: two observed isotopic distributions- (1) natural isotopic distribution, ‘Dnat’; (2) deuterated isotopic distribution, ‘Ddeu’, and one statistical distribution of deteurium contribution to Ddeu, ‘Dlev’. Dlev is expected to conform to the binomial distribution B(*n*, *d*/*n*), where *n* is the number of exchangeable hydrogens in a peptide and *d*, of interest in HDX-MS analysis, is the average number of exchanged deuteriums^9^. The convolution of Dnat and Dlev results in Ddeu, which gets to span a wider *m/z* range than Dnat since Dlev is not a single value but a distribution.

Given Dnat and Ddeu of a peptide, the estimation of *d* is performed as follows: for every possible *d*, theoretical Dlev_*d* is generated and theoretical Ddeu_*d* can be also generated by convoluting Dlev_*d* with Dnat (Let Dlev_# and Ddeu_# be the distributions based on deuterium number #). Then *d* can be determined by finding the best match between any theoretical Ddeu_*d* and the observed Ddeu.

**Figure 4:**
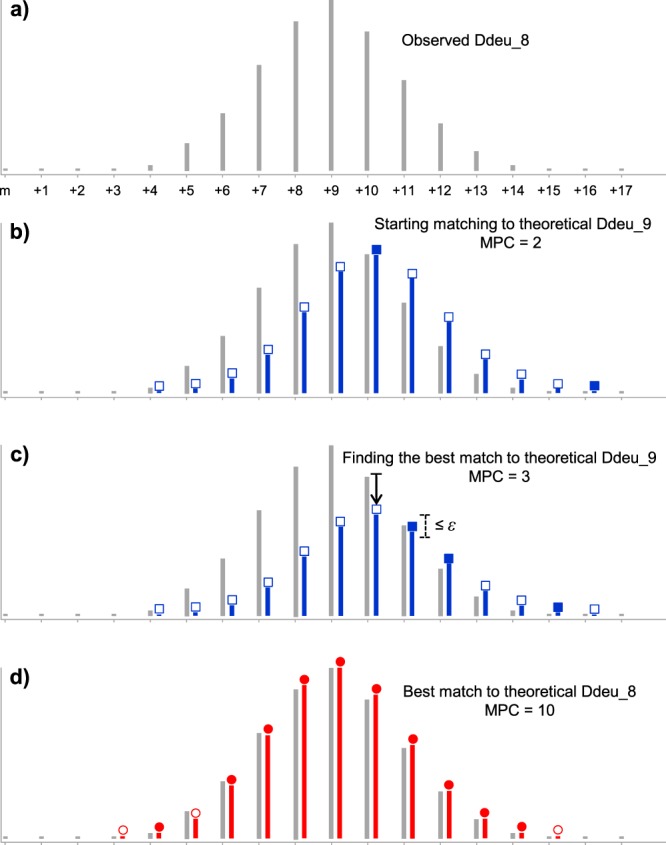
Matched Peak Count (MPC) measures goodness of fit between observed and theoretical distributions. Gray and colored peaks represent experiment and theoretical ones respectively, and the filled figure means that experiment and theoretical peaks are similar (the difference is less than *ε*) in their intensities, increasing MPC value by 1. The tolerance *ε* is defined for each peak as 10% of theoretical peak’s intensity. (**a**) Observed deuterated distribution with average deuterium number 8 is shown. (**b**) Theoretical distribution is first normalized so that its highest peak’s intensity is set to be the same as the observed one at the corresponding position. (**c**) Weight of theoretical distribution is adjusted to find maximum MPC under given average deuterium number. (**d**) Finally, the best fit is found, and then its average deuterium number is determined.

The matching of two distributions is measured by Matched Peak Count (MPC) (eq. 1)

1 $$MPC(O,T)=\sum _{k}M(k),\,M(k)=\{\begin{array}{c}1,\,when|{O}_{k}-N{T}_{k}|\le {\varepsilon }_{k}\\ 0,\,otherwise\end{array}$$

where *O*_*k*_ and *T*_*k*_ are intensity of the *k*-th peak in the observed Ddeu, *O*, and the theoretical Ddeu, *T*, respectively. *N* is a normalization factor to scale the peak intensities in *T* and is first calculated from the position *p* of the most abundant peak in *T* so that *NT*_*p*_ is set equal to *O*_*p*_ as shown in Fig. 4b (i.e., *N* = *O*_*p*_/*T*_*p*_). *ε*_*k*_ is an intensity tolerance in matching *k*-th peak and is set to 10% of *NT*_*k*_ to allow wider tolerance for higher intensity peak. Then for each *d*, MPCs are calculated while varying *N* from 110% to 10% of its initial value in 5% decrement and the maximum MPC is determined as the matching quality for *d* under consideration. In Fig. 4c, for example, the maximum MPC is found when *N* = 0.75 in case *d* = 9. By iteratively performing this process for every *d*, the optimal *d* is determined with maximum MPC (when *d* = 8 in Fig. 4d).

**Figure 5:**
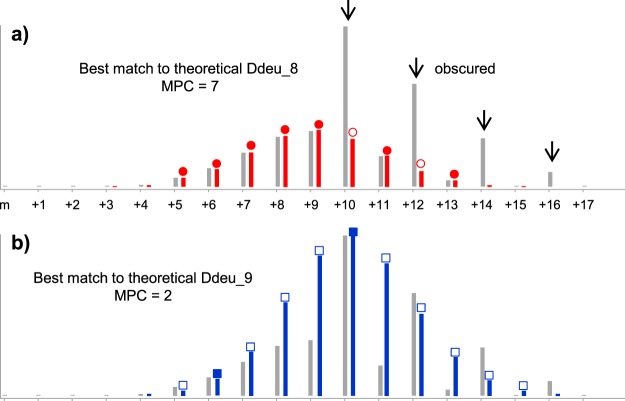
MPC robustness to noisy spectral data. (**a**) One deuterated distribution is obscured by the other distribution at arrow-labeled positions. MPC determines the exact deuterium number, 8. (**b**) MPC value for wrong interpretation is very low. But, chi-square fit does not distinguish the exact number from the wrong one.

Maximizing MPC is somewhat different from minimizing errors in matching distributions. MPC has strength in cases when an experimental distribution has big noises or is obscured by isotopic distribution of other species as shown in Fig. 5. Centroid mass calculation approaches can easily result in a wrong deuterium number in such cases, because the big noises are included in the calculation (thus the centroid mass is shifted to the right in an example shown in Fig. 5). It is also evident that error measurement approaches such as least-square error easily fail for such cases. In the example of Fig. 5, chi-square error of Ddeu_9 was less than that of Ddeu_8 and resulted in the wrong deuterium number, while MPC clearly distinguished the two and determined the exact deuterium number, 8. In addition to robustness to noises, we argue that MPC has great potential to discover a bimodal distribution.

### Toward analyzing bimodal deuterated distributions

**Figure 6:**
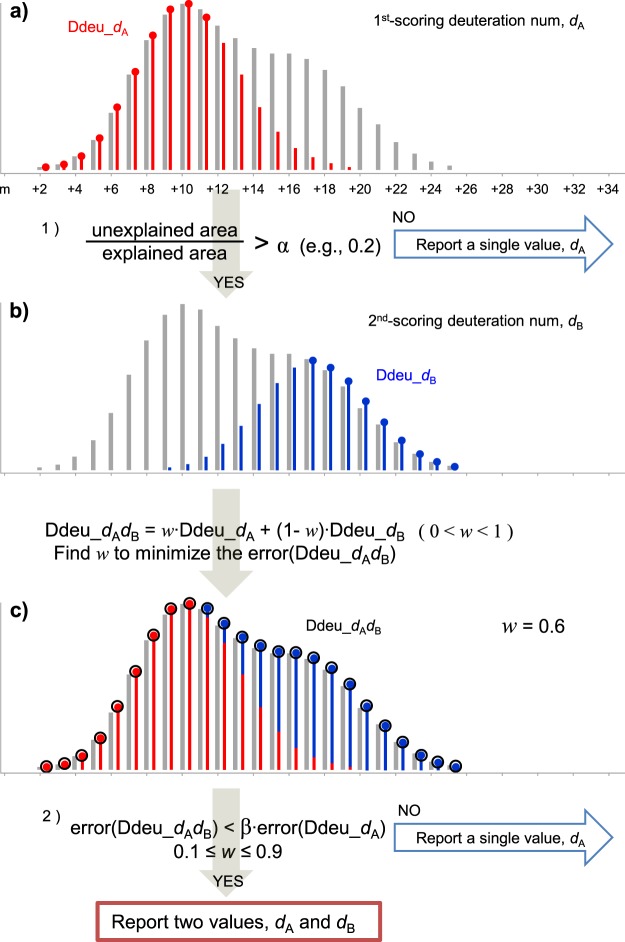
Bimodal distribution analysis. (**a**) After the initial unimodal distribution analysis, it is assessed how well the observed distribution is explained. (**b**) If a significant portion is not explained, the next bimodal distribution analysis is performed, where weights of the two distributions are optimized. (**c**) After bimodal distribution analysis, it is assessed how much the error is improved and whether both distributions are all fairly abundant. Only if all criteria are satisfied, two deuterium numbers are reported.

Figure 6 shows the bimodal analysis process by deMix. The first analysis is conducted assuming a unimodal distribution and results in a single deuterium value, *d*_A_ (Fig. 6a). If the ratio of explained area by the single value to unexplained area does not exceed a certain preset threshold (5:1 in this work), deMix proceeds to bimodal distribution analysis. deMix takes the 2nd-ranked deuterium number, *d*_B_ from the first analysis (Fig. 6b), generates a theoretical bimodal distribution using the two numbers, *d*_A_ and *d*_B_, and compares it with the observed Ddeu. In the bimodal distribution analysis, the problem is to determine how each species is populated in the bimodal distribution (eq. 2).

2 $$\mathrm{Ddeu}\_{d}_{{\rm{A}}}{d}_{{\rm{B}}}=w\cdot \mathrm{Ddeu}\_{d}_{{\rm{A}}}+(1-w)\cdot \mathrm{Ddeu}\_{d}_{{\rm{B}}}$$

deMix finds the optimal *w* using maximum MPC. Finally, deMix reports two deuterium numbers, *d*_A_ and *d*_B_, only if the error of the bimodal distribution analysis is significantly improved than that of the unimodal analysis (because the error of the bimodal distribution analysis is almost always less than that of the unimodal analysis given a higher degree of freedom, its significance should be assessed). In addition, if the weight factor for more abundant species is over 90%, only the abundant one is reported (not accepted as a bimodal distribution).

Basically, for the analysis of the bimodal distribution, all possible combinations of two deuterium numbers need to be examined, but this would be computationally expensive. MPC can successfully select candidates of the two numbers by using non-overlapped area (e.g., right tail in Fig. 6a) in the bimodal distribution. However, if the two numbers are similar and thus, the length of non-overlapped tail is short, MPC may fail to determine the numbers exactly. We found in case that the distance between the two numbers is small, MPC often reported t*d*_A_ plus 1 for a small true value t*d*_A_, while t*d*_B_ minus 1 for a large true value t*d*_B_. To compensate for such off-by-one misinterpretations, deMix in the bimodal analysis takes into account 4 combinations, either *d*_A_−1 or *d*_A_ and either *d*_B_ or *d*_B_+1 and determines the final two values from the combination with the best MPC. We performed simulation tests to show how well MPC predicts two numbers in a bimodal distribution according to the distance between the two numbers. The simulation test was performed as follows: for each peptide of length *n* that was randomly selected from Swiss-Prot human database, two deuterium numbers and their respective weights (the sum of weights should be one) were randomly generated, and theoretical, bimodal deuterated distribution was generated using eq. 2. Then, we checked how correctly MPC predicts the two numbers from the bimodal distributions. In Table 1, each column represents the distance (2, 3, 5, and 8) between the two deuterium numbers and each row represents the weight of a more abundant deuterated form. For each cell, 10,000 trials (or different bimodal distributions) were conducted and the figures show the percentage of times when MPC correctly determines both simulated deuterium numbers. In ‘initial’ column, MPC performance was assessed only by initial prediction *d*_A_ and *d*_B_, while ‘expanded’ column shows the performance when considering *d*_A_ + 1 and *d*_B_ − 1 as well as *d*_A_ and *d*_B_ (*d*_A_ < *d*_B_). In ‘initial’ column, the performance drop was observed when the distance between two component numbers is close or a weight of one component is much higher than the other (e.g., 0.9 vs. 0.1). The performance was worse as the peptide length was longer, where a certain bimodal distribution was very similar to a unimodal distribution and it was difficult to distinguish the two. On the other hand, in ‘expanded’ column considering 4 combinations, MPC showed almost perfect performance in all cases without considering all possible combinations of two deuterium numbers. With high accuracy, the MPC-based calculation was fast and the running time of deMix was a few minutes for a set of HDX experiments (i.e., in processing 7 mzXML files and calculating deuterium numbers from different D_2_O labeling times in this work) on a desktop computer.

**Table 1:** Simulation test on bimodal deuterated distributions.

Distance between two deuterated numbers in a bimodal distribution								
Weight	2		3		5		8	
	Initial	Expanded	Initial	Expanded	Initial	Expanded	Initial	Expanded
**(a) For peptides of length 10**								
0.5	49%	100%	80%	100%	100%	100%	100%	100%
0.6	68%	100%	83%	100%	98%	100%	100%	100%
0.7	37%	100%	86%	100%	92%	100%	100%	100%
0.8	24%	100%	58%	100%	91%	100%	100%	100%
0.9	18%	100%	42%	98%	92%	100%	100%	100%
**(b) For peptides of length 15**								
0.5	54%	100%	79%	100%	100%	100%	100%	100%
0.6	45%	100%	85%	100%	100%	100%	100%	100%
0.7	23%	100%	71%	100%	100%	100%	100%	100%
0.8	18%	100%	53%	100%	100%	100%	100%	100%
0.9	19%	100%	43%	100%	89%	94%	100%	100%
**(c) For peptides of length 20**								
0.5	48%	100%	75%	100%	100%	100%	100%	100%
0.6	29%	100%	74%	100%	100%	100%	100%	100%
0.7	21%	100%	54%	100%	99%	99%	100%	100%
0.8	18%	100%	45%	100%	99%	100%	100%	100%
0.9	18%	100%	42%	100%	83%	97%	100%	100%
**(d) For peptides of length 30**								
0.5	14%	100%	67%	100%	99%	100%	100%	100%
0.6	21%	100%	56%	100%	99%	100%	100%	100%
0.7	19%	100%	47%	100%	97%	98%	100%	100%
0.8	17%	100%	43%	100%	87%	94%	100%	100%
0.9	24%	100%	42%	100%	75%	92%	100%	100%

With varying distances and weights between two distributions in bimodal analysis, deMix performance is shown. Each cell shows how deMix correctly determined two deuterated forms for bimodal distributions simulated under a specific condition. For example, in b), peptides of length 15 were randomly selected from Swiss-Prot human database, and then two deuterium numbers and their respective weights were randomly generated. Based on the simulated values, bimodal deuterated distributions were generated (10,000 different distributions for each cell). When the distance between two deuterium numbers was close, deMix often reported t*d*_A_ plus 1 for a small true value t*d*_A_ while t*d*_B_ minus 1 for a large true value t*d*_B_ and failed to determine the exact numbers (initial column). Based on the observation, deMix in bimodal analysis takes into account 4 combinations using *d*_A_ − 1 and *d*_B_ + 1 in addition to *d*_A_ and *d*_B_ from initial prediction. The adaptation led to outstanding performance for all cases (expanded column). In ‘weight’ row, the weight (*w*) of more abundant form is represented (the weight of the other form is 1-*w*).

### Application to Nm23-H1

Nm23-H1, a tumor metastasis suppressor, is a multifunctional housekeeping enzyme. It is known that NDP kinase activities and metastasis suppressor activities of Nm23-H1 are regulated by redox balance at Cys109^19,20^. Recent study characterized molecular mechanism of this redox regulation as stepwise oxidations. Firstly, under oxidative conditions, Nm23-H1 forms intra-disulfide bond between Cys4 and Cys145, which induces large conformational changes. Secondly, these conformational changes induce oxidations at Cys109 by regulating the quaternary structure^21^. The crystal structures of native Nm23-H1 are available^22^ and recent study has also reported the crystal structure of oxidized Nm23-H1^21^. In this study, HDX experiments for native and oxidized (treated with H_2_O_2_) Nm23-H1 were used to elucidate the conformational changes, and deMix was applied to analyze the data.

Table 2 summarizes the peptides from Nm23-H1 and their HDX analysis results. Higher deuteration rates were observed in the oxidized form than the native form. Especially, the peptide (residue 109–132) containing Cys109 showed dominant difference in its HDX rates between the two conditions. Under the oxidative condition, the intra-disulfide bond induces a conformational change of the C-terminal domain (residue 133–141), which triggers helix-to-loop transition of α8 in the C-terminal domain and thereby exposes Cys109 to be easily accessible to solvent molecules. deMix results clearly showed that under an oxidative condition the HDX rates were increased at both early and saturated points of D_2_O labeling time. In recent study, two interface regions of Nm23-H1 subunits were detected to show a large difference in HDX rate depending on H_2_O_2_ concentration^21^. In this study, kinetics of HDX rate changes in control and 1 mM H_2_O_2_-treated protein were examined. HDX rates in oxidized Nm23-H1 were increased in dimeric interface (residues 9~35) and most of the K-pn loop region (residues 96~108), HDX rates of dimeric interface (9~35) in response to H_2_O_2_ treatment showed gradual increase and then reached saturation depending on D_2_O labeling time. On the other hand, HDX rates of K-pn loop region (residues 96~108) were immediately saturated at the first time point. These kinetic results provide the detail structural information of oxidized Nm23-H1, where K-pn loop in oxidized Nm23-H1 is exposed to surface and dimeric interface is presumed to be more inside than K-pn loop, thus shows a slower change in HDX rate.

**Table 2:** HDX analysis results of Nm23-H1.

m/z	rt (m)	peptide	sites	D_2_O labeling times (Native)							D_2_O labeling times (Oxidative)						
				30 s	1 m	3 m	5 m	10 m	30 m	60 m	30 s	1 m	3 m	5 m	10 m	30 m	60 m
420.69 (2+)	5.96	ANCERTF	2~8	1	1	1	1	1	1~2	2~1	1	1	1	1	1	2	2
566.99 (3+)	8.71	FIAIKPDGVQRGLVGE	8~23	1	1	1	1	1	1	2~0	1~3	2~0	1~3	1~4	3~1	3	4
776.45 (2+)	8.01	IAIKPDGVQRGLVGE	9~23	1	1	1	1	1	1	1	2	2	1~4	1~4	4~1	2~5	5~2
517.97 (3+)	8.01	IAIKPDGVQRGLVGE	9~23	1	2~0	1	1~3	1	2~0	1	1~4	1~4	1~4	1~4	4~1	4	4
663.64 (4+)	10.55	IAIKPDGVQRGLVGEIIKRFEQKG	9~32	0~2	0~3	2	0~3	0~3	2~0	2	2	3~0	1~4	1~4	1~4	2~5	4
767.70 (4+)	11.96	IAIKPDGVQRGLVGEIIKRFEQKGFRL	9~35	×	1	1	1	1~3	×	1	3~0	4~0	1~5	1~5	1~5	2~6	3~8
512.31 (3+)	8.01	IIKRFEQKGFRL	24~35	0~2	1	1	1	1	0~2	1	1~0	1	1	1	1	2~0	1~3
490.31 (2+)	9.84	FRLVGLKF	33~40	0~1	0~1	0~1	1~0	1~0	0~1	1~0	0~1	1~0	1~0	1~0	1	1	1~3
941.48 (3+)	10.2	MQASEDLLKEHYVDLKDRPFFAGL	41~64	7	8	9	9	10	11	11	10	10	10	11	11	11	11
569.81 (4+)	10.2	DLLKEHYVDLKDRPFFAGL	46~64	6	6	7	7	8	8	9~8	8	8	8	8	9	9	9
602.99 (3+)	9.14	LKEHYVDLKDRPFF	48~61	3	3	4	4	4	4	4	4	4	4	4	4	5	4
452.49 (4+)	9.14	LKEHYVDLKDRPFF	48~61	3	3	4	4	4	4	4	4	4	4	4	4	4	4
1024.55 (2+)	9.49	LKEHYVDLKDRPFFAGL	48~64	5	5	6	6	6	6	6	6	6	6	7	6	7	7
559.96 (3+)	10.13	HYVDLKDRPFFAGL	51~64	5	6	6	6	6	7	6~7	6	6	6	7	7	7	7
650.86 (2+)	7.3	LVKYMHSGPVVA	64~75	1	1	1	1~2	2~1	2	2	2~1	2~1	2~1	2	2	2	2
716.38 (2+)	8.08	LVKYMHSGPVVAM	64~76	1	1	1	1	2	2	2	2~1	2	2~1	2	2	2	2
594.32 (2+)	6.59	VKYMHSGPVVA	65~75	2~0	2~0	2~0	2~0	2	2	2	2~0	2~0	2	2	2	2	2
680.85 (2+)	8.08	PADSKPGTIRGDF	96~108	2	2	2	2	3	3	3	3	3	3	3	3	3	3
852.10 (3+)	10.55	CIQVGRNIIHGSDSVESAEKEIGL	109~132	5	5	5	5	6	7	7	7	8	8	9	10	11	12
586.28 (2+)	9.77	WFHPEELVD	133~141	1~3	1~2	1~2	1~2	1~2	2	2	2	2	2	2	2	3	3

Each cell represents the deuteration numbers of input peptides under each condition, where ‘x’ means that deuterated distribution is not detected and #~# means that a peptide is observed as two deuterated forms (more abundant form precedes). “rt (m)” column shows a retention time of a peptide in minutes and “sites” column represents the start and end positions of a peptide in Nm23-H1.

**Figure 7:**
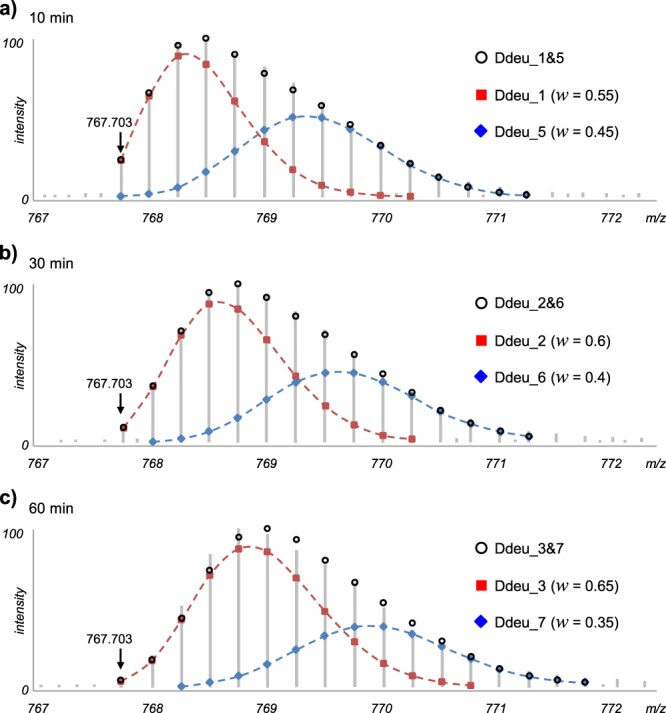
Deuterated distributions of the peptide ‘IAIKPDGVQRGLVGEIIKRFEQKGFRL’ from the oxidized protein at the same retention time with different D_2_O labeling times. The distributions could not be fitted properly using a unimodal distribution, and deMix analyzed them as bimodal forms, whose theoretical distributions (shown as black circle) are well-fitted to their observed distributions. *w* represents relative amount of each component peptide species (shown as red rectangle and blue diamond).

Interestingly, we observed the mixed deuteration behaviors indicating two conformational states for peptides of 9–35 region, a dimeric interface of the protein, presumably resulting from incompletely dissociated dimers in response to H_2_O_2_ treatment. Since Nm23-H1 is regulated by stepwise oxidation, it is expected that intermediate conformations of Nm23-H1 oligomeric states may be observed. Figure 7 shows the mass spectral isotopic distributions from the peptide at *m*/*z* 767.7027 [M+4 H]^4+^ in its oxidized form. The deuterated distributions could not be properly fitted when a single distribution is assumed. For example, the distribution of Fig. 7a was first interpreted as 4 exchanged deuteriums with MPC value 6 and only 62% of observed distribution could be explained. However, bimodal distribution analysis led to 97% explanation of observed distribution with MPC value 12. We observed simultaneous and progressive mass shifts in two components of a bimodal distribution with increasing D_2_O labeling time, and this is different from a phenomenon by EX1 kinetics. It indicates the co-existence of two protein conformations, where one form is specified by the distribution from the exchanged deuterium number 1 to 3 with different D_2_O labeling time, while the other from 5 to 7.

To demonstrate the performance of deMix, we compared with Hexicon 2^17^, an automated pipeline for HDX analysis that supports bimodal analysis like deMix. The overall analysis results of Nm23-H1 by Hexicon 2 agreed with those by deMix. Between the two algorithms, the difference lies in a peptide feature (or isotopic distribution) detection. deMix dynamically determined the elution time spans for candidate peptide masses and aggregated isotopic distributions of the same peptide feature over elution time as shown in Fig. 2 while Hexicon 2 used a single isotopic distribution. We emphasize here the strength of deMix arising from the difference. Hexicon 2 generated two incompatible H/D exchange profiles at different elution times for a single peptide ‘PADSKPGTIRGDF’ (residues 96~108), where one exchange rate increased (consistent with deMix analysis in Table 2) with increasing D_2_O labeling time but the other decreased (wrong by manual validation of the scan, see Supplementary Fig. S1). Hexicon 2 also did not generate an H/D exchange profile for a peptide ‘CIQVGRNIIHGSDSVESAEKEIGL’ (residues 109~132) under oxidative condition. We found that in the two cases, their isotopic signals were weak or noisy. Notably, deMix could generate results for those cases since the aggregation of isotopic distributions over elution time increased the signal-to-noise ratio.

### Visualization software

**Figure 8:**
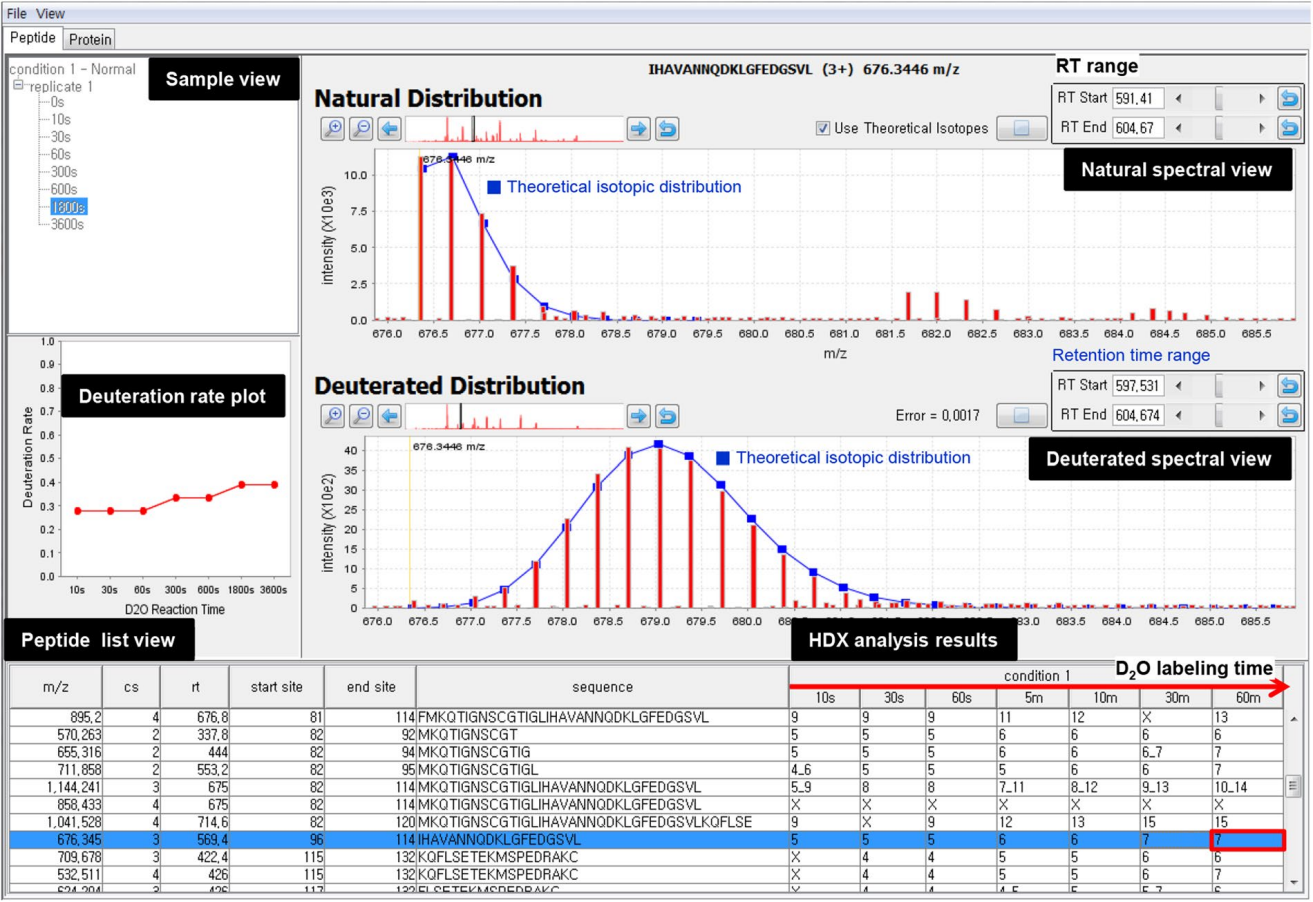
Graphical user interface of deMix visualization tool.

deMix provides visualization software to help users validate and represent HDX analysis results. Figure 8 shows a user-friendly graphical interface of the tool. For spectral data view, two windows are shown: one (upper) is for a natural isotope distribution and the other (lower) for the corresponding deuterated isotope distribution in one of HDX experiments across D_2_O labeling times, which can be selected by a user from a list at the bottom. Peptide list view at the bottom shows input peptides and their HDX analysis results. By selecting a specific cell representing a deuterium number from this list, experimental (red peaks) natural and deuterated isotopic distributions for the peptide can be displayed in the spectral view window together with its theoretical (blue line) distributions, and a deuteration rate plot across D_2_O labeling times is drawn automatically in the left panel. Users can zoom in and out from isotopic distributions in each individual scan, and examine aggregated distributions by adjusting retention time range.

## Discussion

There have been many efforts to characterize protein structures that have the key to elucidating the complex protein function and dynamics. Recently, protein structural changes by oxidation of peroxiredoxin 2^23^, by calcium binding in EF-hands of secretagogin^24^, and by chemical binding to Nm23-H1^25^ and by drug binding to PPARγ^26^ have all been well characterized by employing HDX-MS. HDX-MS is a one of the hottest techniques, which costs less effort and money, and at the same time, it has less limitation compared to other methods such as NMR or X-ray crystallography as described previously^27^. With the growing data size and the technology, a fully automated method is essential for high throughput analysis. deMix takes raw MS data of before and after HDX experiment and their peptide identifications as its input, and analyze the number of exchanged deuteriums. As most of experimental data contain many unexpected noise or co-eluted peptides, deMix effectively addresses lots of nosy peaks during its analysis.

In particular, deMix is very suitable for analyzing bimodal distributions by EX1 kinetics or from two protein conformations, which may suggest an important clue about protein function and dynamics. In the bimodal distribution analysis, the important issue is filtering out false positives. In some cases, a combination of two species may give better fit to the observed distribution than one species, but this may well result from over fitting or from the fact that the original MS data includes noises. For example, the bimodal distribution of Ddeu_6 and Ddeu_8 can be very similar to Ddeu_7, and both unimodal and bimodal analyses generate good results with very small errors. The selection of bimodal distributions over unimodal one must be assessed more conservatively.

## Methods

### Sample Preparation

Recombinant Nm23-H1 protein was purified from *E. coli* strain BL21 (DE3) overexpressing plasmids pET3c containing Nm23-H1 as described previously^28^. Briefly, cytosolic fraction of *E. coli* strains BL21 (DE3) transformed with pET-3c expression plasmids containing Nm23-H1 coding region were obtained after inducing the expression of protein with 0.2 mM IPTG. Each cytosolic fraction was applied to 2~4 mL of ATP-sepharose column equilibrated with Buffer A (20 mM Tris-acetate, 20 mM NaCl, 0.1 mM EDTA, 3 mM MgCl_2_, pH 7.4) at a flow rate of 3 mL/min. The column was then washed with buffer A and then with Buffer A containing 0.25 M NaCl to remove nonspecifically binding proteins. Then Nm23-H1 was eluted with Buffer A containing 1 mM ATP.

### HDX using nanoUPLC-ESI-q-TOF

Recombinant Nm23-H1 (2 μg/μL) was diluted 10-fold with 99% D_2_O for 30 sec, 1, 3, 5, 10, 30, and 60 min and maintained at 25 °C with 1 mM H_2_O_2_. The labeling reaction was quenched by 5 mM tris(2-carboxyethyl)phosphine hydrochloride, pH 2.3 (This is titrated with formic acids). For peptic digestion, porcine pepsin (1 mg/mL) was added to each quenched protein sample and incubated at 0 °C for 3 min before injection^21^.

Deuterated peptic peptides were desalted on line prior to separation using trap column (ID 180 μm × 20 mm, Symmetry® C18) cartridge. Peptides were separated using a C18 reversed-phase 100 μm ID × 100 mm analytical column (1.7 μm particle size, BEH130 C18, Waters Co. USA) with integrated electrospray ionization PicoTipTM (±10 μm ID, New Objective, USA). The auto-sampler chamber was set at 5 °C. The trap, analytical column and all tubing were immersed in an ice bath to minimize deuterium back-exchange. Both mobile phase bottles were placed on ice and both mobile phases contained 0.1% FA. Gradient chromatography was performed at 600 nL/min flow rate and was sprayed on line to mass spectrometer (SYNAPT^TM^ HDMS^TM^, Waters Co. USA). All mass spectral measurements were taken at: capillary voltage 2.5 kV, cone voltage 35 V, extraction cone voltage 4.0 V, source temperature 80 °C. TOF mode scan was performed in range of *m*/*z* 300–1500 with scan time of 1 sec.

### MS/MS-based peptide identification

The raw files were converted to mzXML format using masswolf (v1.4) and were searched by Mascot (version 2.2.07, Matrix Science) with the following parameters: no enzyme option, ±0.3 Da mass tolerance for peptide and fragment ions, and variable modifications of Gln → pyro-Glu (N-terminal Q) and Oxidation (M). Swiss-Prot Human database was used for search. The peptide identifications were obtained at default significant threshold (p < 0.05), only matched to the target protein (Swiss-Prot Number P15531).

### Natural and deuterated peptide profile

deMix’s input consists of a series of mzXML files, one for non-deuterated and the rest from HDX experiments across different D_2_O labeling times, together with the information (sequence, *m*/*z*, charge state, and precursor selection time) of peptides identified from the same sample. For each peptide given, its retention time range is automatically defined first from non-deuterated sample as shown in Fig. 2, and then deuterated isotopic distributions are detected from HDX experiments using the defined retention time range.

To define the retention time range of a peptide, the algorithm examines whether the peptide has been eluted in any of the preceding and/or following MS scans starting from its input precursor selection time, by comparing the peptide’s experimental isotopic distribution with its theoretical isotopic distribution calculated from its sequence information. As a default, the algorithm retrieves around at least ± 40 scans from the precursor selection time, but continues the extension as long as the peptide is detected. The scan extension is terminated if the peptide is not detected in two consecutive MS scans, and then the range along with the center of this elution (CE) is reported (i.e. center of extracted ion chromatogram, XIC), where the center of elution area was calculated as intensity-weighted average time of extended scans. Next, the corresponding deuterated isotopic distribution is determined for HDX experiments, where the shape of its deuterated distribution is not predictable because a peptide’s deuteration number is not known. Thus the algorithm selects the distribution (i.e. a set of peaks) with the maximum consecutive peaks (at least 5) at possible isotopic positions in the HDX-MS scan corresponding to CE, from which scan extension within defined XIC range begins as described above and is finished if in an extended scan, a distribution is not detected or a detected distribution is not overlapped by at least 5 peaks with the distribution at CE. For the deuterated distribution, all distributions detected in individual scans are aggregated together since the aggregated distribution is regarded more robust over errors than individual ones.

In this work, peaks were found within 0.1 Da mass tolerance. A user can set various parameters (default range of scan extension, number of required peaks for distributions, threshold for each peak’s intensity, etc) according to the MS-instruments of choice.

### deMix Software

deMix software suite was implemented in Java programming language (v1.7), and can be obtained upon request to the corresponding author.

## Supplementary information

Supplementary Information

## Data Availability

The authors declare to make the data used in this manuscript available anytime on requirement.
