# Supplementary material for: deMix: Decoding Deuterated Distributions from Heterogeneous Protein States via HDX-MS
Source: Sci Rep. 2019 Feb 28;9:3176. doi: 10.1038/s41598-019-39512-8 (PMC6395696; doi:10.1038/s41598-019-39512-8)
Supplement: Supplementary file 1 — Supplementary Information [file 41598_2019_39512_MOESM1_ESM.pdf]

# Supplementary Information

# deMix: Decoding Deuterated Distributions from Heterogeneous Protein States via HDX-MS

Seungjin Na[1], Jae-Jin Lee[2], Jong Wha J. Joo[3], Kong-Joo Lee[2], and Eunok Paek[1]*

[1]Dept. of Computer Science, Hanyang University, Seoul 04763, South Korea, [2]Graduate School of Pharmaceutical Sciences, College of Pharmacy, Ewha Womans University, Seoul 03760, South Korea, [3]Dept. of Computer Science and Engineering, Dongguk University-Seoul, Seoul 04620, South Korea

To whom correspondence should be addressed: Eunok Paek {eunokpaek@hanyang.ac.kr}

**Supplementary Figure S1.**

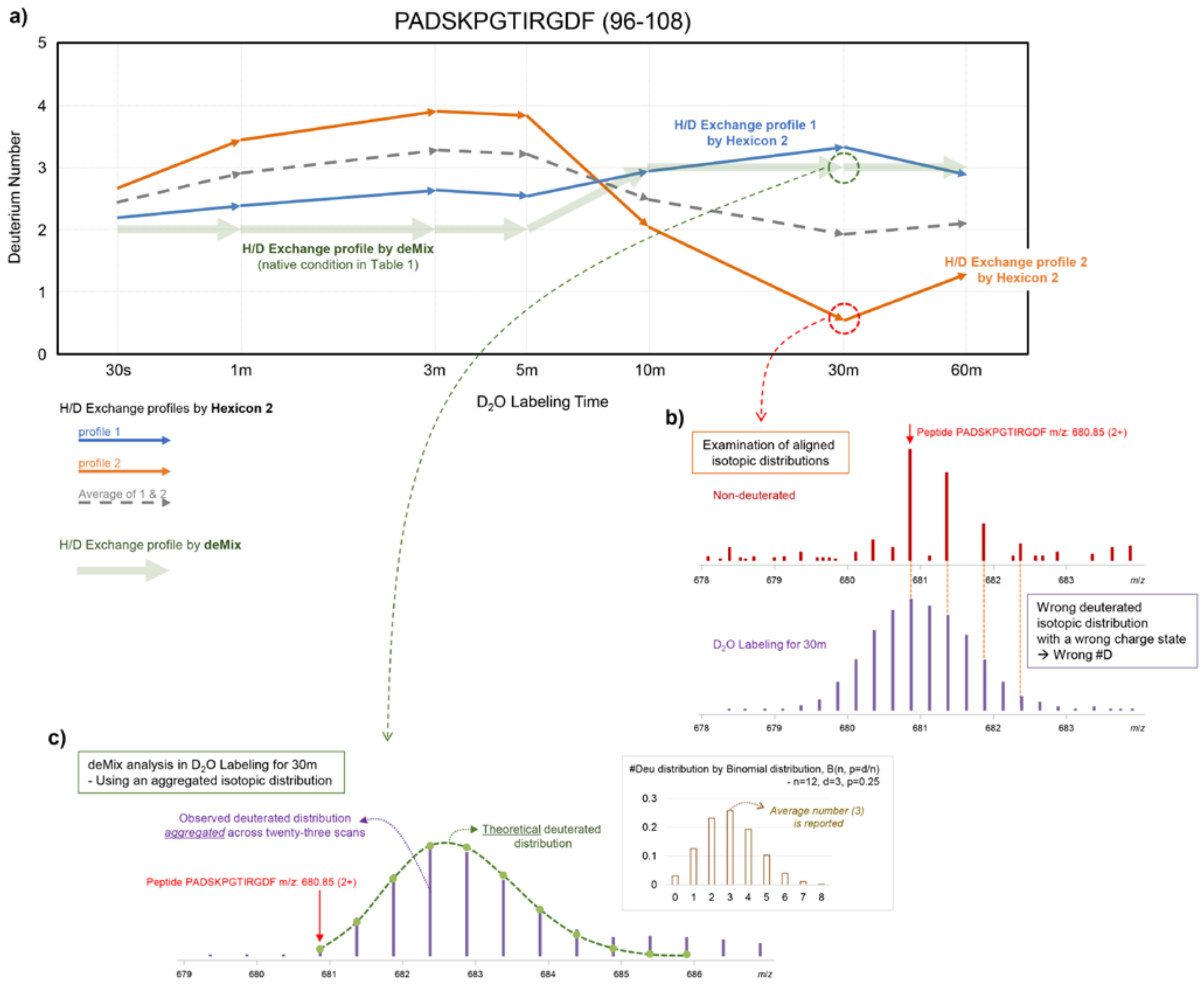


**Supplementary Figure S1.** a) Hexicon 2 showed incompatible H/D exchange results (blue and orange lines) for a peptide 'PADSKPGTIRGDF'. b) The exchange profile 2 (orange line) turned out to be wrong. The charge state of non-deuterated distribution (red peaks) was 2+, while 4+ (by peak spacing) for the aligned deuterated distribution (violet peaks). Different isotopic distributions of 2+ and 4+ charge states overlap in their isotopic peaks as shown in the figure if *m*/*z* values of any peaks are matched. c) deMix uses an aggregated isotopic distribution over elution time and in the figure violet peaks were aggregated from twenty-three scans. The aggregation prevents misleading analysis due to intervention of overlapped or noisy isotopic peaks. deMix interprets deuteration level as binomial distribution (inset) and reports the average number as the final deuterium number (3 in the figure).
